# A cross-sectional survey investigating the burden of, and psychosocial factors related to, psychological distress among people living with HIV in central Tanzania

**DOI:** 10.1186/s40359-025-03813-7

**Published:** 2025-12-17

**Authors:** Tiffany E. Gooden, Jacktan J. Ruhighira, Zainab Mashombo, Janet Mtenga, Emily Kansigo, Paul Sarumbo, Aneth Ngailo, Sheila Greenfield, Semira Manaseki-Holland, G. Neil Thomas, Krishnarajah Nirantharakumar, Stephen Kibusi, Mkhoi L. Mkhoi

**Affiliations:** 1 Department of Applied Health Sciences, University of Birmingham, Birmingham, UK; 2 Department of Medical Physiology, University of Dodoma, Dodoma, Tanzania; 3 Dodoma Regional Referral Hospital, Care and Treatment Center, Dodoma, Tanzania; 4 Makole Health Center, Care and treatment Center, Dodoma, Tanzania; 5 International Center for AIDS program (ICAP), Dar Es Salaam, Tanzania; 6 Department of Public Health, University of Dodoma, Dodoma, Tanzania; 7 Department of Microbiology and Parasitology, University of Dodoma, Dodoma, Tanzania

**Keywords:** HIV, Tanzania, Mental health, Psychological distress, Transdiagnostic factors

## Abstract

**Background:**

Psychological distress is a transdiagnostic factor associated with various mental health conditions which are highly prevalent among people living with HIV (PLWH). The aim of this study was to identify the burden of, and psychosocial factors related to, psychological distress among PLWH in Dodoma, Tanzania.

**Methods:**

A cross-sectional study was conducted among PLWH aged ≥ 18 years recruited consecutively from two healthcare facilities, in person (currently in care) and by phone (lost to care). A questionnaire was interviewer-administered comprising the Kessler Psychological Distress 6 Scale (K6) and questions regarding psychosocial factors including physical health, money/finances, employment/work, family/friends, stigma/disclosure and other HIV-related factors. Logistic regression was used to calculate adjusted odds ratios (aOR) of distress for key sub-groups using evidence-based thresholds from the K6. Differences in psychosocial factors related to distress were investigated using multivariate regression. Regressions were adjusted for age, sex, tribe, employment status, education level, relationship status, residential type (urban/rural), care status, number of children, duration of living with HIV and study site.

**Results:**

461 PLWH participated in the study; 67% were aged ≥ 36 years, 71% were female and 58% were currently in care. More than half of PLWH (*n* = 270; 59%) were experiencing mild to severe distress; with 52% mildly distressed. PLWH employed by someone and PLWH self-employed had lower odds of distress compared to unemployed PLWH (aORs of 0.30, 95% CI 0.14–0.65 and 0.32, 95% CI 0.16–0.64, respectively). PLWH in a relationship also had lower odds of distress compared to PLWH not in a relationship (aOR 0.43, 95% CI 0.28–0.67). PLWH diagnosed > ten years ago had significantly higher odds of having psychological distress compared to PLWH diagnosed < five years prior (aOR 1.97, 95% CI 1.14–3.41). Physical health, money/finances and family/friends were associated with distress; however, factors differed between study sites, age, residential type, employment, relationship status and care status.

**Conclusions:**

A concerningly high proportion of PLWH are suffering from mild to severe distress which can manifest into depression, anxiety, substance misuse or other mental health conditions. Further investigations are needed to understand the interaction between psychosocial factors and distress and how distress can be prevented and reduced.

**Supplementary Information:**

The online version contains supplementary material available at 10.1186/s40359-025-03813-7.

## Introduction

HIV is often inextricably intertwined with social and psychological adversities. Such adversities are common in the general population but elevated among people living with HIV (PLWH) including social isolation, financial, employment, housing, and food insecurities, inadequate social networks, physical and sexual abuse, and extreme poverty [1, 2]. PLWH also experience high levels of stigma related to HIV [3]. Like people with other chronic conditions, PLWH also have to cope with daily medications, regular healthcare appointments, symptoms and side effects, all of which can create chronic psychological distress [4]. 

Chronic psychological distress is a transdiagnostic mechanism related to various mental health conditions such as depression, anxiety, substance use disorders and suicide [5, 6]. PLWH with depression, anxiety or substance use disorders are less likely to adhere to antiretroviral therapy (ART) which increases the risk of forward transmission, opportunistic infections, comorbidities and death [7–9]. Thus, preventing mental health conditions and reducing symptoms among PLWH is of public health and individual importance.

Over 25 million PLWH live in sub-Saharan Africa (65% globally) where various negative psychosocial factors are elevated such as chronic poverty and stigma [10]. Guidelines in low-and middle-income countries (LMICs) often provide guidelines on management of depression and anxiety among PLWH but lack formal recommendations for addressing psychological distress to prevent such conditions. In Tanzania for instance, guidelines recommend psychoeducation, supporting counselling or pharmacological interventions for those experiencing anxiety or major depressive disorder [11]. However, using evidence-based interventions to target psychological distress could prevent the onset of, or reduce symptom severity for, anxiety and depression.

Taking a transdiagnostic approach of targeting psychological distress to prevent mental health conditions may be beneficial for resource-limited settings [12]. Such an approach has the potential to reach more people, use fewer resources, address coexisting mental health conditions, prevent and reduce mental health symptoms, and avoid stigmatisation related to mental health [12]. Understanding the local burden and causes of distress is crucial for advocating for the development of a transdiagnostic intervention to be tested and evaluated for national scale up.

One study conducted in Tanzania reported psychological distress among 21.7% of participants recruited from the general population [13]. However, evidence on the burden of psychological distress among PLWH is lacking. Evidence that does exist varies from setting to setting. For instance, a 2023 meta-analysis of 15 studies found that the prevalence of psychological distress among PLWH was 44%, ranging from 6%-79% [14]. None of the studies reviewed were conducted in Tanzania where an estimated 1.8 million PLWH live and around 43,000 new diagnoses are made each year [15]. Given the variation in burden reported among existing studies, local evidence is imperative to understand whether a targeted intervention for PLWH would be useful.

Given the scarcity of evidence, we aimed to determine the burden of, and psychosocial factors related to, psychological distress among PLWH in Central Tanzania. Secondly, we aimed to determine whether the burden of psychological distress and associated factors differ among sub-groups of PLWH, and the risk factors for moderate to severe psychological distress.

## Materials and methods

### Study design and setting

This cross-sectional study was conducted from July to December 2023 in Dodoma, Tanzania. Dodoma is the capital city of Tanzania with an estimated 765,000 people; however, the wider Dodoma area is mostly rural and includes nearly three million people. Located in Central Tanzania, Dodoma City is the governmental hub of the country and has five hospitals, 13 health centres and the only mental health hospital in the country (Mirembe National Mental Health Hospital). Participants were recruited from CTCs located at Dodoma Regional Referral Hospital and Makole Healthcare Centre. These CTCs combined have the largest number of PLWH registered across the wider Dodoma region (*n* > 9000). At the time of the study, HIV care was provided free to all PLWH across Tanzania including ART, viral load tests and CD4 tests.

### Study participants

We aimed to recruit PLWH retained in care and PLWH lost to care. PLWH retained in care were eligible if they were aged 18 years or older and attending the care and treatment centre (CTC) for HIV care. PLWH lost to care were eligible if they were aged 18 years or older, had missed their previous healthcare appointment for HIV care and had not transferred their HIV care to another CTC. As requested by local healthcare staff, anyone who received an HIV diagnosis less than two weeks prior to recruitment was excluded to avoid interference with the counselling and tailored care offered to people newly diagnosed.

With the assumption that 44% of PLWH will be experiencing mild to severe psychological stress [14] and considering a 10% decline rate, a minimum sample of 422 PLWH was required for a ± 5 precision.

### Study procedures

Three CTC healthcare professionals from Dodoma Regional Referral Hospital and two from Makole Healthcare Centre attended a one-day training on recruitment, obtaining consent, and data collection. All five healthcare professionals were also involved in developing the methods and data collection tool to ensure culturally and contextually accepted methods and terminology were used. The healthcare professionals were instructed to assess eligibility for each PLWH they see in care and each PLWH lost to care they call. PLWH were recruited consecutively, from Monday to Friday until the required sample size was met. PLWH lost to care were called as per normal practice whereby a list of PLWH who missed their last appointment is printed each day and each person is then phoned to assess why they missed their appointment and to try to get them to return to care. All PLWH called were assessed for eligibility and eligible PLWH were subsequently recruited following consent.

A questionnaire comprised of 38 questions (supplementary material) was administered to all eligible and consented PLWH either in person or by phone. The first 19 questions were demographic and care characteristic questions. This included information on age, sex, tribe, employment status, marital status, number of children, care status, whether they lived in a rural or urban setting and duration of living with HIV. Care status was determined based on the point of recruitment; participants recruited from the CTC in person were considered in care and participants recruited over the phone were considered lost to care. Participants were asked which area of Dodoma they reside in, and the study team then determined whether their residence met the criteria for rural or urban based on government definitions.

Questions 20–26 consisted of a validated short-form assessment questionnaire for psychological distress, the Kessler Psychological Distress 6 scale (K6) [16]. The K6 has been used and validated in Swahili and for use in Tanzania [17] and other African settings [18, 19]. Psychological distress is assessed by six unidimensional items using a 5-point Likert scale; options are provided for participants to select how often they felt nervous, hopeless, restless/fidgety, so depressed that nothing could cheer them up, that everything was an effort, and worthless in the previous 30 days all of the time, most of the time, some of the time, a little of the time or none of the time. Questions 27–34 assessed how often the participants’ distress was due to issues regarding their (1) physical health, (2) money/finances, (3) employment/work, (4) family/friends, (5) stigma/disclosure, (6) other HIV-related factors, and/or (7) any other reasons not listed. These psychosocial factors were determined through stakeholder consultation with CTC clinicians, CTC nurses, psychiatrists, community health workers and PLWH. Only participants who said they had experienced any distress from questions 20–26 were asked questions 27–34; these questions were in the same format as questions 20–26 whereby a Likert scale was used to determine whether each factor/issue was the main cause of distress all of the time, most of the time, some of the time, a little of the time or none of the time. The final four questions asked the participants about their opinions on the need and want for a course on stress reduction and their interest in being involved in a patient and public involvement group for developing and implementing such a course. All data was collected on paper and entered into an Excel sheet within 48 h of data collection.

### Outcomes

The primary outcome was the prevalence of psychological distress overall and for the following sub-groups: CTC clinics, age groups, sex, tribe, employment status, education level, marital status, urban or rural inhabitant and care status. Each of the six items from the K6 are scored from 0 to 4 where none of the above is a score of 0 and all of the time is a score of 4; the scores are then summed to provide an overall score representing severity of distress [16]. With a possible score range of 0–24, the prevalence of distress was calculated based on evidence that a score of 0 represents no distress, scores 1–7 represent mild distress, scores 8–12 represent moderate distress and 13–24 represent severe distress [16, 20]. 

Secondary outcomes included risk factors for, and psychosocial factors related to, moderate to severe distress. The following were investigated as possible risk factors (all categorical): age, sex, tribe, employment status, education level, marital status, whether they live in an urban or rural area and their care status. We categorised tribes based on the region of origin; regional tribes tend to be similar in traditions and culture. Psychosocial factors were scored similar to the K6 questions where each category was given a score of 0–4; a score of 0 indicated that a particular category did not contribute at all to the distress experienced in the previous 30 days.

### Analysis

Stata version 17 was used for analysis. Descriptive statistics were used to present participant demographic and clinical characteristics and prevalence of distress, presenting median and interquartile range (IQR) for continuous data and frequency and percentages for categorical data. Unadjusted and adjusted logistic regression models were used to present odds ratios with 95% confidence intervals (CI) for moderate to severe distress among each sub-group of PLWH. An adjusted multivariate regression model was used to determine the psychosocial factors related with moderate to severe distress where for each factor investigated, individual scores of 1–4 were compared to a score of 0.

Covariates in all adjusted models included age, number of children and duration of living with HIV (in years), CTC clinics, sex, tribe, employment status, education level, relationship status, residential type (whether they live in an urban or rural area) and their care status (in care or lost to care) as categorical variables. Covariates were selected based on existing literature [14] and discussion with CTC healthcare staff. Collinearity was examined using variance inflation factors (VIFs) from a linear regression model including the same covariates as the logistic regression analyses. All VIFs were ≤ 5, except for one age category (26–35 years; VIF = 5.74). Given that age is an important confounder and the VIF value was only marginally elevated, age was retained in the model. These findings indicate no problematic multicollinearity among the included variables.

Recognising that odds ratios can overestimate relative measures when outcomes are common, we conducted a sensitivity analysis using a modified Poisson regression with robust (sandwich) standard errors to estimate adjusted prevalence ratios (aPRs) [21]. 

This study is best conceptualised as a stratified sample rather than clustered data due to recruitment from only two clinics. Standard approaches for cluster adjustment (e.g., cluster-robust standard errors or multilevel models) are not appropriate or reliable with only two clusters [22] and sampling weights were unnecessary given the nearly equal numbers recruited from each clinic. To account for site-level differences regarding availability of mental health services and case mix, we calculated prevalence estimates and odds ratios stratified by site in a post-hoc analysis. All statistical tests were two-sided with a p-value of ≤ 0.05 considered statistically significant.

## Results

### Participant characteristics

A total of 461 PLWH participated in the study, 42% of which were considered lost to care at the time of data collection (Table 1). Most participants were aged 36 years or older (67%), female (71%), from a central zone tribe (61%), lived in an urban setting (81%), were self-employed (61%) and had 1–3 children (60%). 58% were on ART at the time of data collection which aligns with the proportion considered lost to care. Just over half were in a relationship (55%). Participants had been living with HIV for a mean of 6.7 years, with nearly half living with HIV for less than five years (43%).

**Table 1:** Participant characteristics

	Dodoma Regional Referral Hospital			Makole Health Centre			All participants		
	In care *n* = 142	Lost to care *n* = 83	Total *n* = 225	In care *n* = 127	Lost to care *n* = 109	Total *n* = 236	In care *n* = 269	Lost to care *n* = 192	Total *n* = 461
Age									
18–25	6 (4.2)	14 (16.9)	20 (8.9)	4 (3.2)	10 (9.2)	14 (5.9)	10 (3.7)	24 (12.5)	34 (7.4)
26–35	21 (14.8)	18 (21.7)	39 (17.3)	33 (26.0)	33 (30.3)	66 (28.0)	54 (20.1)	51 (26.6)	105 (22.8)
36–45	39 (27.5)	29 (34.9)	68 (30.2)	39 (30.7)	38 (34.9)	77 (32.6)	78 (29.0)	67 (34.9)	145 (31.5)
46–55	37 (26.1)	12 (14.5)	49 (21.8)	32 (25.2)	22 (20.2)	54 (22.9)	69 (25.7)	34 (17.7)	103 (22.3)
56+	7 (8.4)	31 (21.8)	38 (16.9)	17 (13.4)	4 (3.7)	21 (8.9)	48 (17.4)	11 (5.7)	59 (12.8)
Missing	3 (3.6)	8 (5.6)	11 (4.9)	2 (1.6)	2 (1.8)	4 (1.7)	10 (3.7)	5 (2.6)	15 (3.3)
Sex									
Female	109 (76.8)	53 (63.9)	162 (72.0)	97 (76.4)	70 (64.2)	167 (70.8)	206 (76.6)	123 (64.1)	329 (71.4)
Male	33 (23.2)	30 (36.1)	63 (28.0)	29 (22.8)	39 (35.8)	68 (28.8)	62 (23.1)	69 (35.9)	131 (28.4)
Missing	0 (0)	0 (0)	0 (0)	1 (0.8)	0 (0)	0 (0)	1 (0.4)	0 (0)	0 (0)
Tribe									
Central zone	80 (56.3)	51 (61.5)	131 (58.2)	83 (65.4)	65 (59.6)	148 (62.7)	163 (60.6)	116 (60.4)	279 (60.5)
Southern zone	26 (18.3)	5 (6.0)	31 (13.8)	15 (11.8)	20 (18.4)	35 (14.8)	41 (15.2)	25 (13.0)	66 (14.3)
Western zone	0 (0)	1 (1.2)	1 (0.4)	1 (0.8)	3 (2.8)	4 (1.7)	1 (0.4)	4 (2.1)	5 (1.1)
Eastern zone	23 (16.2)	4 (4.8)	27 (12.0)	11 (8.7)	7 (6.4)	18 (7.6)	34 (12.6)	11 (5.7)	45 (9.8)
Northern zone	5 (3.5)	9 (10.8)	14 (6.2)	6 (4.7)	9 (8.3)	15 (6.4)	11 (4.1)	18 (9.4)	29 (6.3)
Lake zone	8 (5.6)	12 (14.5)	20 (8.9)	10 (7.9)	5 (4.6)	15 (6.4)	18 (6.7)	17 (8.9)	35 (7.6)
Missing	0 (0)	1 (1.2)	1 (0.4)	1 (0.8)	0 (0)	1 (0.4)	1 (0.4)	1 (0.5)	2 (0.4)
Residential type									
Urban	114 (80.3)	67 (80.7)	181 (80.4)	108 (85.0)	86 (78.9)	194 (82.2)	222 (82.5)	153 (79.7)	375 (81.3)
Rural	28 (19.7)	16 (19.3)	44 (19.6)	19 (15.0)	19 (17.4)	38 (16.1)	47 (17.5)	35 (18.2)	82 (17.8)
Missing	0 (0)	0 (0)	0 (0)	0 (0)	4 (3.7)	4 (1.7)	0 (0)	4 (2.1)	4 (0.9)
Employment									
Unemployed	17 (12.0)	30 (36 (1)	47 (20.9)	14 (11.0)	18 (16.5)	32 (13.6)	31 (11.5)	48 (25.0)	79 (17.1)
Employed by someone	23 (16.2)	16 (19.3)	39 (17.3)	23 (18.1)	38 (34.9)	61 (25.9)	46 (17.1)	54 (28.1)	100 (21.7)
Self-employed	101 (71.1)	37 (44.6)	138 (61.3)	90 (70.9)	51 (46.8)	141 (59.8)	191 (71.0)	88 (45.8)	279 (60.5)
Missing	1 (0.7)	0 (0)	1 (0.4)	0 (0)	2 (1.8)	2 (0.9)	1 (0.4)	2 (1.0)	3 (0.7)
Relationship status									
In a relationship	63 (44.4)	38 (45.8)	101 (44.9)	69 (54.3)	82 (75.2)	151 (64.0)	132 (49.1)	120 (62.5)	252 (54.7)
Not in a relationship	79 (55.6)	45 (54.2)	124 (55.1)	58 (45.7)	27 (24.8)	85 (36.0)	137 (50.9)	72 (37.5)	209 (45.3)
Number of children									
No children	16 (11.3)	14 (16.9)	30 (13.3)	10 (7.9)	15 (13.8)	25 (10.6)	26 (9.7)	29 (15.1)	55 (11.9)
1–3 children	79 (55.6)	49 (59.0)	128 (56.9)	75 (59.1)	73 (67.0)	148 (62.7)	154 (57.3)	122 (63.5)	276 (59.9)
4 + children	46 (32.4)	17 (20.5)	63 (28.0)	41 (32.3)	20 (18.4)	61 (25.9)	87 (32.3)	37 (19.3)	124 (26.9)
Missing	1 (0.7)	3 (3.6)	4 (1.8)	1 (0.8)	1 (0.9)	2 (0.9)	2 (0.7)	4 (2.1)	6 (1.3)
ART status									
On ART	141 (99.3)	2 (2.4)	143 (63.6)	124 (97.6)	1 (0.9)	125 (53.0)	265 (98.5)	3 (1.6)	268 (58.1)
Not on ART	1 (0.7)	81 (97.6)	82 (36.4)	2 (1.6)	107 (98.2)	109 (46.2)	3 (1.1)	188 (97.9)	191 (41.4)
Missing	0 (0)	0 (0)	0 (0)	1 (0.8)	1 (0.9)	2 (0.9)	1 (0.4)	1 (0.5)	2 (0.4)
Duration of living with HIV									
Mean (SD)	8.8 (5.2)	5.4 (5.2)	7.6 (5.4)	7.4 (5.7)	4.1 (4.5)	5.9 (5.5)	8.1 (5.5)	4.7 (4.9)	6.7 (5.5)
< 5 years	27 (19.0)	47 (56.6)	74 (32.9)	54 (42.5)	72 (66.1)	126 (53.4)	81 (30.1)	119 (62.0)	200 (43.4)
5–10 years	51 (35.9)	11 (13.3)	62 (27.6)	26 (20.5)	19 (17.4)	45 (19.1)	77 (28.6)	30 (15.6)	107 (23.2)
> 10 years	62 (43.7)	22 (26.5)	84 (37.3)	46 (36.2)	16 (14.7)	62 (26.3)	108 (40.2)	38 (19.8)	146 (31.7)
Missing	2 (1.4)	3 (3.6)	5 (2.2)	1 (0.8)	2 (1.8)	3 (1.3)	3 (1.1)	5 (2.6)	8 (1.7)

Compared to the proportion retained in care, the following groups of PLWH had a notable higher proportion lost to care: younger (aged 18–45 years), male, unemployed, employed by someone, in a relationship, 0–3 children, and living with HIV for less than five years. Several differences were found between the two CTCs. There was a higher proportion of PLWH aged 56 years or more, unemployed, not in a relationship, on ART, and living with HIV for five or more years from Dodoma Regional Referral Hospital whereas there was a higher proportion of PLWH in a relationship, not on ART, and living with HIV for less than five years from Makole Health Centre.

### Burden of psychological distress

The mean distress score was 2.2 (SD 2.9) with 59% (*n* = 270) of PLWH experiencing mild to severe psychological distress (Table 2), 89% of which were experiencing mild distress (*n* = 241/270) and less than 1% (*n* = 5/270) were experiencing severe distress.

**Table 2:** Prevalence of psychological distress overall and by key sub-groups

	Mean distress score Mean (SD)	Prevalence of psychological distress				Unadjusted odds ratio (95% CI) for moderate to severe scores	Adjusted odds ratio (95% CI) for moderate to severe scores
		None *N* (%)	Mild *N* (%)	Moderate *N* (%)	Severe *N* (%)		
Overall	2.2 (2.9)	191/461 (41.4)	241/461 (52.3)	24/461 (5.2)	5/461 (1.1)	--	--
CTC facility							
Dodoma Regional Referral Hospital	2.8 (3.2)	67/225 (29.8)	140/225 (62.2)	14/225 (6.2)	4/225 (1.8)	Ref	Ref
Makole Health Centre	1.6 (2.6)	124/236 (52.5)	101/236 (42.8)	10/236 (4.2)	1/236 (0.4)	0.38 (0.26–0.56)*	0.46 (0.30–0.70)*
Sex							
Female	2.3 (2.9)	130/329 (39.5)	178/329 (54.1)	18/329 (5.5)	3/329 (0.9)	Ref	Ref
Male	2.0 (3.0)	60/131 (45.8)	63/131 (48.1)	6/131 (4.6)	1/131 (1.5)	0.77 (0.51–1.16)	0.88 (0.55–1.41)
Age							
18–25	2.8 (2.4)	8/34 (23.5)	25/34 (73.5)	1/34 (2.9)	0/34 (0)	Ref	Ref
26–35	2.0 (2.8)	52/105 (49.5)	46/105 (43.8)	7/105 (6.7)	0/105 (0)	0.31 (0.13–0.76)*	0.39 (0.13–1.16)
36–45	2.0 (2.7)	62/145 (42.8)	75/145 (51.7)	7/145 (4.8)	1/145 (0.7)	0.41 (0.17–0.97)*	0.51 (0.17–1.55)
46–55	2.0 (2.4)	45/103 (43.7)	54/103 (52.4)	4/103 (3.9)	0/103 (0)	0.40 (0.16–0.96)*	0.42 (0.14–1.32)
56+	3.2 (3.9)	16/59 (27.1)	35/59 (59.3)	5/59 (8.5)	3/59 (5.1)	0.83 (0.31–2.20)	0.47 (0.13–1.65)
Residential type							
Rural	1.8 (2.5)	37/82 (45.1)	42/82 (51.2)	3/82 (3.7)	0/82 (0)	Ref	Ref
Urban	2.3 (3.0)	153/375 (40.8)	196/375 (52.3)	21/375 (5.6)	5/375 (1.3)	1.19 (0.74–1.93)	1.32 (0.76–2.29)
Employment							
Unemployed	3.1 (3.0)	15/79 (19.0)	59/79 (74.7)	4/79 (5.1)	1/79 (1.3)	Ref	Ref
Employed by someone	1.8 (2.5)	49/100 (49.0)	46/100 (46.0)	5/100 (5.0)	0/100 (0)	0.24 (0.12–0.48)*	0.30 (0.14–0.65)*
Self-employed	2.0 (2.9)	126/279 (45.2)	135/279 (48.4)	15/279 (5.4)	3/279 (1.1)	0.28 (0.15–0.52)*	0.32 (0.16–0.64)*
Relationship status							
Not in a relationship	2.9 (3.2)	60/209 (28.7)	131/209 (62.7)	14/209 (6.7)	4/209 (1.9)	Ref	Ref
In a relationship	1.6 (2.6)	131/252 (52.0)	110/252 (43.7)	10/252 (4.0)	1/252 (0.4)	0.37 (0.25–0.55)*	0.43 (0.28–0.67)*
Number of children							
No children	2.4 (2.5)	20/55 (36.4)	33/55 (60.0)	2/55 (3.6)	0/55 (0)	Ref	Ref
1–3 children	2.2 (3.0)	116/276 (42.0)	140/276 (50.7)	17/276 (6.2)	3/276 (1.1)	0.79 (0.43–1.43)	1.62 (0.73–3.58)
4 + children	2.1 (3.0)	52/124 (41.9)	65/124 (52.4)	5/124 (4.0)	2/124 (1.6)	0.79 (0.41–1.52)	1.38 (0.57–3.34)
Care status							
Lost to care	1.9 (2.2)	78/192 (40.6)	110/192 (57.3)	4/192 (2.1)	0/192 (0)	Ref	Ref
In care	2.4 (3.3)	113/269 (42.0)	131/269 (48.7)	20/269 (7.4)	5/269 (1.9)	0.94 (0.65–1.38)	0.78 (0.49–1.26)
Duration of living with HIV							
< 5 years	1.8 (2.5)	97/200 (48.5)	95/200 (47.5)	7/200 (3.5)	1/200 (0.5)	Ref	Ref
5–10 years	2.0 (2.9)	44/107 (41.1)	57/107 (53.3)	5/107 (4.7)	1/107 (0.9)	1.35 (0.84–2.17)	1.38 (0.80–2.39)
> 10 years	2.8 (3.2)	46/146 (31.5)	86/146 (58.9)	12/146 (8.2)	2/146 (1.4)	2.05 (1.31–3.20)*	1.97 (1.14–3.41)*

*CI* Confidence interval, *SD* Standard deviation, *CTC* Care and treatment centre

*P-value < 0.05

Before and after adjustment for confounders, there were significant differences in psychological distress between the two CTC clinics, employment status, relationship status, and duration of living with HIV. PLWH from Makole Health Centre had lower odds of having psychological distress compared to PLWH from Dodoma Regional Referral Hospital (aOR 0.46, 95% CI 0.30–0.70) and the same was true for PLWH employed by someone or self-employed when compared to unemployed PLWH (aORs of 0.30, 95% CI 0.14–0.65 and 0.32, 95% CI 0.16–0.64, respectively) and PLWH in a relationship compared to PLWH not in a relationship (aOR 0.43, 95% CI 0.28–0.67). PLWH with a duration of living with HIV for more than ten years had significantly higher odds of having psychological distress compared to PLWH diagnosed less than five years prior (aOR 1.97, 95% CI 1.14–3.41).

In the unadjusted model, PLWH aged 26–35, 36–45 and 46–55 years had lower odds of having psychological distress compared to PLWH aged 18–25 years (aORs of 0.31, 95% CI 0.13–0.76; 0.41, 95% CI 0.17–0.97; 0.40, 95% CI 0.16–0.96, respectively); however, this became insignificant in the adjusted model.

The post-hoc analysis indicated similar trends in the mean distress scores among sub-groups stratified by clinic site; however, results lacked precision (supplementary materials Tables 1 and 2). From the adjusted model, there were no significant findings among sub-groups for those recruited from Makole Healthcare Centre. From Dodoma Regional Referral Hospital, those in a relationship remained having significantly lower odds for having moderate to severe distress compared to those in a relationship (aOR 0.47, 95% CI 0.22–0.97). An additional significant finding from this site was those currently in care having significantly higher odds compared to those lost to care (aOR 3.15, 95% CI 1.26–7.91).

Results to the sensitivity analysis are presented in Supplementary material Table 3. The aPRs were generally consistent in direction and significance with the odds ratios; however, employment status nor duration of living with HIV were no longer significant using the Poisson regression. Relationship status remained significant and being in care became statistically significant in this sensitivity analysis. 

### Psychosocial factors related to psychological distress

Mean scores were low for each factor among those with psychological distress (Table 3). However, physical health, money/finances and family/friends contributed toward psychological distress for 46%, 50% and 53% PLWH, respectively (Table 3). Thirty-two percent of PLWH said employment/work contributed to their distress whilst stigma/disclosure and other HIV-related factors were said to contribute to distress among 19% and 17% of PLWH, respectively.

**Table 3:** Mean scores for each psychosocial factor

	Psychosocial factors Mean (SD)					
	Physical health	Money / finances	Employment / work	Family / friends	Stigma / disclosure	Other HIV-related factors
Overall	0.72 (0.9)	0.86 (1.0)	0.53 (0.9)	0.97 (1.1)	0.38 (0.9)	0.28 (0.7)
CTC facility						
Dodoma Regional Referral Hospital	0.77 (0.9)	1.02 (1.1)	0.69 (1.0)	0.78 (1.0)	0.44 (1.0)	0.37 (0.8)
Makole Health Centre	0.65 (1.0)	0.63 (0.9)	0.30 (0.7)	1.23 (1.1)	0.29 (0.7)	0.14 (0.5)
Sex						
Female	0.71 (0.9)	0.85 (1.0)	0.50 (0.9)	0.99 (1.1)	0.36 (0.9)	0.25 (0.7)
Male	0.75 (0.9)	0.86 (1.0)	0.61 (1.0)	0.89 (1.0)	0.44 (1.0)	0.35 (0.8)
Age						
18–25	0.65 (0.9)	0.58 (0.8)	0.73 (1.2)	0.65 (0.9)	1.00 (1.4)	0.23 (0.5)
26–35	0.75 (1.0)	0.89 (1.0)	0.49 (0.8)	0.79 (1.0)	0.45 (1.0)	0.34 (0.8)
36–45	0.61 (0.8)	0.80 (1.0)	0.43 (0.8)	1.02 (1.0)	0.34 (0.8)	0.22 (0.6)
46–55	0.66 (0.9)	1.03 (1.1)	0.60 (1.0)	0.95 (1.1)	0.14 (0.5)	0.24 (0.7)
56+	0.88 (1.1)	0.81 (1.0)	0.42 (0.8)	1.35 (1.2)	0.21 (0.6)	0.37 (0.9)
Residential type						
Rural	0.80 (0.8)	1.09 (1.1)	0.40 (0.7)	1.07 (1.0)	0.31 (0.7)	0.22 (0.5)
Urban	0.70 (1.0)	0.80 (1.0)	0.56 (0.9)	0.94 (1.1)	0.40 (0.9)	0.29 (0.8)
Employment						
Unemployed	0.84 (0.9)	1.13 (1.1)	0.67 (0.9)	0.92 (1.0)	0.31 (0.8)	0.23 (0.6)
Employed by someone	0.69 (0.9)	0.55 (0.9)	0.27 (0.6)	0.76 (1.0)	0.73 (1.2)	0.39 (1.0)
Self-employed	0.65 (0.9)	0.84 (1.0)	0.56 (1.0)	1.05 (1.1)	0.29 (0.8)	0.25 (0.6)
Relationship status						
Not in a relationship	0.81 (1.0)	0.97 (1.1)	0.69 (1.0)	0.92 (1.1)	0.43 (1.0)	0.31 (0.7)
In a relationship	0.60 (0.9)	0.72 (0.9)	0.33 (0.6)	1.02 (1.0)	0.32 (0.8)	0.24 (0.7)
Number of children						
No children	0.69 (0.9)	0.63 (0.8)	0.71 (1.0)	0.89 (1.0)	0.69 (1.2)	0.17 (0.5)
1–3 children	0.81 (1.0)	0.88 (1.0)	0.54 (1.0)	0.93 (1.1)	0.40 (0.9)	0.34 (0.8)
4 + children	0.56 (0.8)	0.94 (1.0)	0.42 (0.7)	1.10 (1.1)	0.21 (0.7)	0.19 (0.6)
Care status						
Lost to care	0.82 (0.9)	0.73 (0.9)	0.32 (0.8)	0.94 (0.9)	0.36 (0.8)	0.25 (0.7)
In care	0.64 (0.9)	0.95 (1.1)	0.67 (1.0)	0.99 (1.1)	0.40 (0.9)	0.30 (0.7)
Duration of living with HIV						
< 5 years	0.79 (1.0)	0.78 (0.9)	0.50 (0.9)	0.83 (1.0)	0.51 (1.0)	0.29 (0.7)
5–10 years	0.71 (0.9)	0.90 (1.1)	0.70 (0.9)	0.94 (1.0)	0.40 (1.0)	0.27 (0.8)
> 10 years	0.63 (0.9)	0.89 (1.0)	0.47 (0.8)	1.15 (1.1)	0.24 (0.7)	0.26 (0.7)

*CI* Confidence interval, *SD* Standard deviation, *CTC* Care and treatment centre

*P-value < 0.05

Some factors differed between CTC clinics, age, residential type, employment status, relationship status and care status (Table 4). Compared to Makole Health Centre, a higher proportion of PLWH from Dodoma Regional Referral Hospital said that physical health (51% vs. 38%), money/finances (56% vs. 41%), employment/work (40% vs. 21%), and other HIV-related factors (22% vs. 10%) contributed to their distress whereas a higher proportion of PLWH from Makole Health Centre said family/friends (65% vs. 44%) contributed to their distress. The proportion of PLWH that said family/friends contributed to distress increased as they increased in age, with 38% of the youngest group (18–25 years) saying this compared to 70% of the older group (56 years or more). Conversely, the proportion of PLWH that said stigma/disclosure contributed to distress decreased with age, with 9% of those aged 46 to 55 years old saying this compared to 42% of those aged 18 to 25 years old.

**Table 4:** Differences in psychosocial factors across sub-groups

	Physical health		Money / finances		Employment / work		Family / friends		Stigma / disclosure		Other HIV-related factors	
	*N* (%)	*p*-value	*N* (%)	*p*-value	*N* (%)	*p*-value	*N* (%)	*p*-value	*N* (%)	*p*-value	*N* (%)	*p*-value
Overall	124/270 (46)	--	134/270 (50)	--	87/270 (32)	--	143/270 (53)	--	51/270 (19)	--	46/270 (17)	--
CTC facility												
DRRH	81/158 (51)	**0.037**	88/158 (56)	**0.018**	63/158 (40)	**0.001**	70/158 (44)	**0.001**	33/158 (21)	0.319	35/158 (22)	**0.008**
MHC	43/112 (38)		46/112 (41)		24/112 (21)		73/112 (65)		18/112 (16)		11/112 (10)	
Sex												
Female	88/199 (44)	0.347	99/199 (50)	0.948	62/199 (31)	0.530	107/199 (54)	0.657	37/199 (19)	0.835	31/199 (16)	0.286
Male	36/71 (51)		35/71 (49)		25/71 (35)		36/71 (51)		14/71 (20)		15/71 (21)	
Age												
18–25	11/26 (42)	0.651	11/26 (42)	0.681	10/26 (39)	0.775	10/26 (38)	**0.034**	11/26 (42)	**0.004**	5/26 (19)	0.943
26–35	23/53 (43)		26/53 (49)		17/53 (32)		23/53 (43)		12/53 (23)		10/53 (19)	
36–45	37/83 (45)		41/83 (49)		24/83 (29)		49/83 (59)		15/83 (18)		12/83 (14)	
46–55	24/58 (41)		33/58 (57)		20/58 (34)		29/58 (50)		5/58 (9)		9/58 (16)	
56+	24/43 (56)		19/43 (44)		11/43 (26)		30/43 (70)		5/43 (12)		8/43 (19)	
Residential type												
Rural	27/45 (60)	**0.040**	26/45 (58)	0.200	12/45 (27)	0.353	27/45 (60)	0.265	8/45 (18)	0.804	8/45 (18)	0.915
Urban	96/222 (43)		105/222 (47)		75/222 (34)		113/222 (51)		43/222 (19)		38/222 (17)	
Employment												
Unemployed	37/64 (58)	0.052	39/64 (61)	**0.013**	27/64 (42)	0.063	35/64 (55)	0.101	10/64 (16)	**0.011**	11/64 (17)	0.972
Employed by someone	24/51 (47)		17/51 (33)		11/51 (22)		20/51 (39)		17/51 (33)		9/51 (18)	
Self-employed	61/153 (40)		76/153 (50)		49/153 (32)		86/153 (56)		23/153 (15)		25/153 (16)	
Relationship status												
Not in a relationship	76/149 (51)	0.063	80/149 (54)	0.139	57/149 (38)	**0.019**	74/149 (50)	0.228	29/149 (19)	0.789	29/149 (19)	0.239
In a relationship	48/121 (40)		54/121 (45)		30/121 (25)		69/121 (57)		22/121 (18)		17/121 (14)	
Number of children												
No children	16/35 (46)	0.333	15/35 (43)	0.546	16/35 (46)	0.162	17/35 (49)	0.412	10/35 (29)	0.073	5/35 (14)	0.183
1–3 children	79/160 (49)		80/160 (50)		50/160 (31)		82/160 (51)		33/160 (21)		33/160 (21)	
4 + children	28/72 (39)		39/72 (54)		20/72 (28)		43/72 (60)		8/72 (11)		8/72 (11)	
Care status												
Lost to care	61/114 (54)	**0.033**	52/114 (46)	0.259	24/114 (21)	**0.001**	65/114 (57)	0.254	21/114 (18)	0.867	18/114 (16)	0.641
In care	63/156 (40)		82/156 (53)		63/156 (40)		78/156 (50)		30/156 (19)		28/156 (18)	
Duration of living with HIV												
< 5 years	51/103 (50)	0.453	51/103 (50)	0.915	30/103 (29)	0.243	49/103 (48)	0.204	25/103 (24)	0.190	19/103 (18)	0.591
5–10 years	30/63 (48)		30/63 (48)		26/63 (41)		33/63 (52)		10/63 (16)		8/63 (13)	
> 10 years	41/100 (41)		51/100 (51)		31/100 (31)		60/100 (60)		15/100 (15)		18/100 (18)	

*CTC* Care and treatment centre, *DRRH* Dodoma Regional Referral Hospital, *MHC* Makole Health Centre

Physical health contributed to distress more among PLWH living in rural areas as compared to urban areas (60% vs. 43%) and PLWH lost to care compared to those retained in care (54% vs. 40%). A lower proportion of PLWH employed by someone said that money/finances contributed to distress compared to PLWH unemployed or self-employed (33% vs. 61% vs. 50%, respectively) whereas the opposite was true for stigma/disclosure (33% vs. 16% vs. 15%, respectively). Compared to PLWH in a relationship, a higher proportion of PLWH not in a relationship said that employment/work contributed to their distress (38% vs. 25%). A higher proportion of PLWH retained in care said employment/work contributed to their distress compared to PLWH lost to care (40% vs. 21%).

## Discussion

We report the burden of, and psychosocial factors related to, psychological distress among PLWH and various sub-groups of PLWH from two CTC clinics in Dodoma, Tanzania. More than half had a score indicative of having mild to severe distress with higher odds occurring among PLWH from Dodoma Regional Referral Hospital, and who were unemployed, not in a relationship or with a longer duration of living with HIV. Physical health, money/finances and family/friends were the leading contributors to distress overall; however, this varied substantially across sub-groups of PLWH such as sex and age.

We report a higher burden of distress than a recent meta-analysis which reported a prevalence of 44% from 15 studies [14]. However, this could be due to the inclusion of six studies from high-income countries, where distress may systematically differ from sub-Saharan Africa, or from the variation of measurement tools used to define distress. Our results are in line with four studies that were conducted in sub-Saharan Africa including studies from Uganda (51%), Ethiopia (73%) and two studies from Nigeria (65%, 73%). Important to note, our results indicate a rate of psychological distress more than twice as high among PLWH compared to the general population in Tanzania (21.7%), as reported by a similar sized study using the K10 scale [13]. 

Overall, 46% of PLWH experiencing distress said physical health issues was a contributor and this raised to 60% among PLWH living in rural areas. This finding may be reflective of the lack of easy access to healthcare for rural inhabitants in LMICs which remains a considerable barrier to care [23, 24]. However, PLWH are at higher risk of developing various physical health conditions and diseases [25, 26]. Thus, distress due to physical health may be elevated among PLWH compared to people without HIV which should be investigated. Money/finances was also said to be a major contributor to distress, but this was more of a distressing factor for PLWH unemployed or self-employed. Evidence from Asia depict many barriers to PLWH securing and sustaining employment such as potential discriminatory practices in the workplace, concerns over health (frequent hospital admissions, physical weakness), and ability to keep HIV status secret from employers and colleagues [27]. Such issues should be explored in Tanzania to determine whether these issues also impact the ability of PLWH to find secure and stable employment in a different setting.

Family/friends was another leading contributor to distress overall and this increased with age. This could be due to many reasons such as the impact of HIV on family dynamics or more generic reasons not specific to HIV. Further research is needed to ascertain what impact living with HIV can have regarding family and friend dynamics in Tanzania. Understanding the complex relationship between PLWH and their family and friends as they age is imperative to know if and how healthcare staff could better support them.

Nineteen percent of PLWH with distress said stigma/disclosure was a major contributor; this is in line with evidence from various regions in Tanzania that have reported non-disclosure among around 20% of PLWH [28–30]. There is a paucity of evidence on whether stigma differs based on age; however, we found that a higher proportion of younger PLWH said stigma/disclosure caused them distress. Whilst one study in South Africa found that a higher proportion of older PLWH experienced stigma, this was not significantly different than younger PLWH [31]. A Canadian study found that PLWH age 50 years and older had significantly lower levels of stigma compared to PLWH age 40 years or younger; a disaggregated analysis indicates that younger PLWH experience higher levels of enacted, anticipated and internalised stigma compared to PLWH above the age of 40 [32]. More work is needed to improve disclosure rates and ensure younger PLWH are supported in overcoming and coping with stigmatisation; indeed, the UNAIDS guide to ending HIV-related stigma and discrimination list adolescent PLWH as a key and vulnerable population to target [33]. 

A major strength of this study is the diverse and well-powered sample with the inclusion of PLWH lost to care. To our knowledge this is the first study to use the K6 among PLWH; however, the tool has been used and validated in Swahili and Tanzania [17]. One main limitation to mention is that we did not include a comparison group of people without HIV; therefore, an association between psychological distress and HIV was not possible. We only sampled PLWH from two clinics located in the capital city; therefore, generalisability to other regions in Tanzania or rural clinics may not be possible. The 2024 UNAIDS data from the United Republic of Tanzania estimates that 61% of PLWH are female which is lower than our sample (71%).^15^ This could be due to gender differences in research participation, healthcare seeking and/or being contactable by phone during working hours. However, our sample composition by age is in line with trends seen in prevalence reported in the 2023 Tanzania HIV Impact Report: the lowest prevalence was reported among women and men aged 15–19 years (0.8% and 0.3%, respectively) and the highest prevalence was reported to be among women aged 40–49 (13.0%) and men aged 50–54 (8.4%) [34].

## Conclusion

We report on the burden and causes of psychological distress among PLWH in Tanzania. Psychological distress can manifest in depression, anxiety or other mental health conditions; thus, it is concerning that we found a high burden of mild to severe distress. However, the burden and causes varied based on sub-groups of PLWH which need further exploring to understand the relationship between psychosocial factors and distress and how living with HIV interacts with this relationship. Nonetheless, prevention and reduction of distress among PLWH must be prioritised to improve quality of life, wellbeing and ART adherence among this vulnerable population.

## Supplementary Information

Supplementary Material 1.

## Data Availability

The datasets used and/or analysed during the current study are available from the corresponding author on reasonable request.

## References

[1] Nanni MG, Caruso R, Mitchell AJ, Meggiolaro E, Grassi L. Depression in HIV infected patients: a review. Curr Psychiatry Rep. 2015;17(1):530. 25413636 10.1007/s11920-014-0530-4

[2] UNAIDS. Seizing the moment: tackling entrenched inequalities to end epidemics. Switzerland: UNAIDS Geneva; 2020.

[3] Rueda S, Mitra S, Chen S, et al. Examining the associations between HIV-related stigma and health outcomes in people living with HIV/AIDS: a series of meta-analyses. BMJ Open. 2016;6(7):e011453. 27412106 10.1136/bmjopen-2016-011453 PMC4947735

[4] Liddy C, Blazkho V, Mill K. Challenges of self-management when living with multiple chronic conditions: systematic review of the qualitative literature. Can Fam Physician. 2014;60(12):1123–33. 25642490 PMC4264810

[5] Wu C-Y, Lee M-B, Liao S-C, Chan C-T, Liu L-yD, Chen C-Y. Psychological distress of suicide attempters predicts one-year suicidal deaths during 2007–2016: A population-based study. J Formos Med Assoc. 2020;119(8):1306–13. 32444260 10.1016/j.jfma.2020.04.033

[6] Lass AN, Winer ES. Distress tolerance and symptoms of depression: A review and integration of literatures. Clin Psychol (New York). 2020;27(3):80.

[7] Tao J, Vermund SH, Qian HZ. Association between depression and antiretroviral therapy use among people living with HIV: A meta-analysis. AIDS Behav. 2018;22(5):1542–50. 28439754 10.1007/s10461-017-1776-8 PMC7942230

[8] Velloza J, Kemp CG, Aunon FM, Ramaiya MK, Creegan E, Simoni JM. Alcohol use and antiretroviral therapy non-adherence among adults living with HIV/AIDS in sub-Saharan africa: a systematic review and meta-analysis. AIDS Behav. 2020;24:1727–42. 31673913 10.1007/s10461-019-02716-0 PMC7190427

[9] Wykowski J, Kemp CG, Velloza J, Rao D, Drain PK. Associations between anxiety and adherence to antiretroviral medications in Low- and Middle-Income countries: A systematic review and Meta-analysis. AIDS Behav. 2019;23(8):2059–71. 30659424 10.1007/s10461-018-02390-8 PMC6639150

[10] World Health Organization. HIV, Data on the size of the HIV/AIDS epidemic. 2024. https://www.who.int/data/gho/data/themes/topics/indicator-groups/indicator-group-details/GHO/number-of-people-(all-ages)-living-with-hiv (accessed 14th November 2024.

[11] National AIDS Control Programme. National guidelines for the management of HIV and AIDS. The united Republic of Tanzania. Ministry of Health, Community Development, Gender, Elderly and Children; 2019. https://hivpreventioncoalition.unaids.org/en/resources/tanzania-national-guidelines-management-hiv-and-aids-7th-edition. Accessed 12 Dec 2025.

[12] Holmes E, Ghaderi A, Harmer C, et al. Psychological treatments research in tomorrow’s science: seeing further. Lancet Psychiatry. 2018;5(3):237–86. 29482764 10.1016/S2215-0366(17)30513-8

[13] Ivanova O, Sineke T, Wenzel R, et al. Health-related quality of life and psychological distress among adults in tanzania: a cross-sectional study. Arch Public Health. 2022;80(1):144. 35610653 10.1186/s13690-022-00899-y PMC9127286

[14] Ma H, Zhu F, Zhai H, et al. Prevalence of psychological distress among people living with HIV/AIDS: a systematic review and meta-analysis. AIDS Care. 2023;35(2):153–64. 35642250 10.1080/09540121.2022.2080802

[15] UNAIDS. United Republic of Tanzania. 2024. (Accessed 3rd Oct 2025. https://www.unaids.org/en/regionscountries/countries/unitedrepublicoftanzania

[16] Yiengprugsawan V, Kelly M, Tawatsupa B. Kessler psychological distress scale. Encyclopedia Qual Life well-being Res. 2014:3469–70. 10.1007/978-94-007-0753-5_3663.

[17] Vissoci JRN, Vaca SD, El-Gabri D, et al. Cross-cultural adaptation and psychometric properties of the Kessler scale of psychological distress to a traumatic brain injury population in Swahili and the Tanzanian setting. Health Qual Life Outcomes. 2018;16(1):1–8. 30053816 10.1186/s12955-018-0973-0 PMC6062865

[18] Van Heyningen T, Honikman S, Tomlinson M, Field S, Myer L. Comparison of mental health screening tools for detecting antenatal depression and anxiety disorders in South African women. PLoS ONE. 2018;13(4):e0193697. 29668725 10.1371/journal.pone.0193697 PMC5906008

[19] Makanjuola VA, Onyeama M, Nuhu FT, Kola L, Gureje O. Validation of short screening tools for common mental disorders in Nigerian general practices. Gen Hosp Psychiatry. 2014;36(3):325–9. 24559789 10.1016/j.genhosppsych.2013.12.010

[20] Kessler RC, Green JG, Gruber MJ, et al. Screening for serious mental illness in the general population with the K6 screening scale: results from the WHO world mental health (WMH) survey initiative. Int J Methods Psychiatr Res. 2010;19(S1):4–22. 20527002 10.1002/mpr.310 PMC3659799

[21] Zou G. A modified Poisson regression approach to prospective studies with binary data. Am J Epidemiol. 2004;159(7):702–6. 15033648 10.1093/aje/kwh090

[22] Cameron AC, Miller DL. A practitioner’s guide to cluster-robust inference. J Hum Resour. 2015;50(2):317–72.

[23] Gizaw Z, Astale T, Kassie GM. What improves access to primary healthcare services in rural communities? A systematic review. BMC Prim Care. 2022;23(1):313. 36474184 10.1186/s12875-022-01919-0 PMC9724256

[24] Dawkins B, Renwick C, Ensor T, Shinkins B, Jayne D, Meads D. What factors affect patients’ ability to access healthcare? An overview of systematic reviews. Trop Med Int Health. 2021;26(10):1177–88. 34219346 10.1111/tmi.13651

[25] Gooden TE, Gardner M, Wang J, et al. Incidence of cardiometabolic diseases in people with and without human immunodeficiency virus in the united kingdom: A population-based matched cohort study. J Infect Dis. 2021;225(8):1348–56. 10.1093/infdis/jiab420 PMC9016421 34417792

[26] Calcagno A, Nozza S, Muss C, et al. Ageing with HIV: a multidisciplinary review. Infection. 2015;43(5):509–22. 25987480 10.1007/s15010-015-0795-5

[27] Tan SY, Ow Yong LM, Foong JYE, Wong NHS, Chew LL, Koh YL. Securing and sustaining employment: concerns of HIV patients in Singapore. Soc Work Health Care. 2013;52(10):881–98. 24255973 10.1080/00981389.2013.827148

[28] Buma D, Bakari M, Fawzi W, Mugusi F. The influence of HIV-status disclosure on adherence, immunological and virological outcomes among HIV-infected patients started on antiretroviral therapy in Dar-es-Salaam, Tanzania. J HIV/AIDS. 2015;1(3). 10.16966/2380-5536.111.

[29] John ME, Chipwaza B. HIV status disclosure among adults attending care and treatment clinic in Kilombero district, South-Eastern Tanzania. Int J Afr Nurs Sci. 2022;17:100434.

[30] Alexander AG, Relf M, Bosworth HB, Mmbaga BT, Muiruri C. Disclosure of HIV Status to Sexual Partners Among People With HIV in Singida Regional Referral Hospital of Tanzania: A Cross-Sectional Study. J Assoc Nurses AIDS Care. 2024;35(5):397–408. 10.1097/JNC.0000000000000486 PMC11346699 39105516

[31] Ncitakalo N, Mabaso M, Joska J, Simbayi L. Factors associated with external HIV-related stigma and psychological distress among people living with HIV in South Africa. SSM Popul Health. 2021;14:100809. 10.1016/j.ssmph.2021.100809. 10.1016/j.ssmph.2021.100809 PMC8121694 34027011

[32] Emlet CA, Brennan DJ, Brennenstuhl S, Rueda S, Hart TA, Rourke SB. The impact of HIV-related stigma on older and younger adults living with HIV disease: does age matter? AIDS Care. 2015;27(4):520–8. 25397643 10.1080/09540121.2014.978734

[33] Joint United Nations Programme on HIV/AIDS. Practical guide to ending HIV-related stigma and discrimination — Best practices and innovative approaches to reduce stigma and discrimination at the country level, 2023.

[34] US President’s Emergency Plan for AIDS Relief (PEPFAR). Tanzania HIV Impact Survey THIS 2022–2023, 2023. https://microdata.nbs.go.tz/index.php/catalog/53. Accessed 12 Dec 2025.

