# Supplementary material for: A cross-sectional survey investigating the burden of, and psychosocial factors related to, psychological distress among people living with HIV in central Tanzania
Source: BMC Psychol. 2025 Dec 17;14:89. doi: 10.1186/s40359-025-03813-7 (PMC12822005; doi:10.1186/s40359-025-03813-7)
Supplement: Supplementary file 1 — Supplementary Material 1. [file 40359_2025_3813_MOESM1_ESM.docx]

Table 1. Burden of distress overall and by sub-groups for PLWH recruited from Dodoma Regional Referral Hospital

|  | Mean distress score  Mean (SD) | Prevalence of psychological distress | | | | Unadjusted odds ratio (95% CI) for moderate to severe scores | Adjusted odds ratio (95% CI) for moderate to severe scores |
| --- | --- | --- | --- | --- | --- | --- | --- |
|  |  | None  N (%) | Mild  N (%) | Moderate  N (%) | Severe  N (%) |  |  |
| Overall | 2.8 (3.2) | 67/225 (29.8) | 140/225 (62.2) | 14/225 (6.2) | 4/225 (1.8) | -- | -- |
| Sex |  |  |  |  |  |  |  |
| Female | 2.9 (3.1) | 44/162 (27.2) | 105/162 (64.8) | 11/162 (6.8) | 2/162 (1.2) | Ref | Ref |
| Male | 2.6 (3.4) | 23/63 (36.5) | 35/63 (55.6) | 3/63 (4.8) | 2/63 (3.2) | 0.72 (0.35-1.48) | 1.47 (0.62-3.51) |
| Age |  |  |  |  |  |  |  |
| 18-25 | 3.6 (2.5) | 2/20 (10.0) | 17/20 (85.0) | 1/20 (5.0) | 0/20 (0.0) | Ref | Ref |
| 26-35 | 2.9 (2.8) | 11/39 (28.2) | 25/39 (64.1) | 3/39 (7.7) | 0/39 (0.0) | 0.59 (0.19-1.83) | 1.21 (0.25-5.77) |
| 36-45 | 2.5 (3.0) | 23/68 (33.8) | 40/68 (58.8) | 4/68 (5.6) | 1/68 (1.5) | 0.29 (0.10-0.87)* | 0.46 (0.09-2.25) |
| 46-55 | 2.5 (2.6) | 16/49 (32.7) | 30/49 (61.2) | 3/49 (6.1) | 0/49 (0.0) | 0.34 (0.11-1.07) | 0.42 (0.08-2.17) |
| 56+ | 3.2 (4.0) | 10/38 (26.3) | 23/38 (60.5) | 3/38 (7.9) | 2/38 (5.3) | 0.54 (0.17-1.69) | 0.35 (0.06-1.88) |
| Residential type |  |  |  |  |  |  |  |
| Rural | 2.6 (2.9) | 16/44 (36.4) | 25/44 (56.8) | 3/44 (6.8) | 0/44 (0.0) | Ref | Ref |
| Urban | 2.9 (3.2) | 51/181 (28.2) | 115/181 (63.5) | 11/181 (6.1) | 4/181 (2.2) | 0.76 (0.36-1.60) | 0.58 (0.24-1.36) |
| Employment |  |  |  |  |  |  |  |
| Unemployed | 3.6 (3.4) | 6/47 (12.8) | 37/47 (78.7) | 4/47 (6.4) | 1/47 (2.1) | Ref | Ref |
| Employed by someone | 2.5 (2.7) | 13/39 (33.3) | 23/39 (59.0) | 3/39 (7.7) | 0/39 (0.0) | 0.71 (0.27-1.87) | 0.61 (0.19-1.96) |
| Self-employed | 2.6 (2.9) | 48/138 (34.8) | 80/138 (58.0) | 8/138 (5.8) | 2/138 (1.5) | 0.60 (0.28-1.27) | 0.42 (0.16-1.10) |
| Relationship status |  |  |  |  |  |  |  |
| Not in a relationship | 3.3 (3.3) | 28/124 (22.6) | 85/124 (68.6) | 8/124 (6.5) | 3/124 (2.4) | Ref | Ref |
| In a relationship | 2.3 (3.0) | 39/101 (38.6) | 55/101 (54.5) | 6/101 (5.9) | 1/101 (1.0) | 0.46 (0.24-0.89)* | 0.47 (0.22-0.97)* |
| Number of children |  |  |  |  |  |  |  |
| No children | 3.5 (2.7) | 6/30 (20.0) | 22/30 (73.3) | 2/30 (6.7) | 0/30 (0) | Ref | Ref |
| 1-3 children | 2.8 (3.2) | 37/128 (28.9) | 80/128 (62.5) | 9/128 (7.0) | 2/128 (1.6) | 0.35 (0.15-0.81)* | 0.38 (0.12-1.22) |
| 4+ children | 2.7 (3.4) | 22/63 (34.9) | 36/63 (57.1) | 3/63 (4.8) | 2/63 (3.2) | 0.31 (0.12-0.80)* | 0.37 (0.10-1.41) |
| Care status |  |  |  |  |  |  |  |
| Lost to care | 2.4 (2.4) | 25/83 (30.1) | 55/83 (66.3) | 3/83 (3.6) | 0/83 (0.0) | Ref | Ref |
| In care | 3.1 (3.5) | 42/142 (29.6) | 85/142 (59.9) | 11/142 (7.8) | 4/142 (2.8) | 1.80 (0.91-3.57) | 3.15 (1.26-7.91)* |
| Duration of living with HIV |  |  |  |  |  |  |  |
| <5 years | 2.7 (2.8) | 21/74 (28.4) | 48/74 (64.9) | 4/74 (5.4) | 1/74 (1.4) | Ref | Ref |
| 5-10 years | 2.3 (3.1) | 23/62 (37.1) | 35/62 (56.5) | 3/62 (4.8) | 1/62 (1.6) | 0.76 (0.31-1.83) | 0.75 (0.27-2.11) |
| >10 years | 3.2 (3.2) | 22/84 (26.2) | 54/84 (64.3) | 7/84 (8.3) | 1/84 (1.2) | 1.67 (0.80-3.48) | 2.36 (0.92-6.06) |

Table 2. Burden of distress overall and by sub-groups for PLWH recruited from Makole Healthcare Centre

|  | Mean distress score  Mean (SD) | Prevalence of psychological distress | | | | Unadjusted odds ratio (95% CI) for moderate to severe scores | Adjusted odds ratio (95% CI) for moderate to severe scores |
| --- | --- | --- | --- | --- | --- | --- | --- |
|  |  | None  N (%) | Mild  N (%) | Moderate  N (%) | Severe  N (%) |  |  |
| Overall | 1.6 (2.6) | 124/236 (52.5) | 101/236 (42.8) | 10/236 (4.2) | 1/236 (0.4) | -- | -- |
| Sex |  |  |  |  |  |  |  |
| Female | 1.7 (2.6) | 86/167 (51.5) | 73/167 (43.7) | 7/167 (4.2) | 1/167 (0.6) | Ref | Ref |
| Male | 1.5 (2.5) | 37/68 (54.4) | 28/68 (41.2) | 3/68 (4.4) | 0/68 (0.0) | 0.63 (0.20-1.98) | 1.22 (0.34-4.32) |
| Age |  |  |  |  |  |  |  |
| 18-25 | 1.6 (1.6) | 6/14 (42.9) | 8/14 (57.1) | 0/14 (0.0) | 0/14 (0.0) | Ref | Ref |
| 26-35 | 1.5 (2.7) | 41/66 (62.1) | 21/66 (31.8) | 4/66 (6.1) | 0/66 (0.0) | 0.71 (0.17-3.04) | 2.15 (0.33-14.05) |
| 36-45 | 1.5 (2.4) | 39/77 (50.7) | 35/77 (45.5) | 3/77 (3.9) | 0/77 (0.0) | 0.51 (0.12-2.23) | 1.23 (0.22-6.90) |
| 46-55 | 1.4 (2.2) | 29/54 (53.7) | 24/54 (44.4) | 1/54 (1.9) | 0/54 (0.0) | 0.35 (0.07-1.91) | 0.86 (0.13-5.76) |
| 56+ | 3.0 (3.8) | 6/21 (28.6) | 12/21 (57.1) | 2/21 (9.5) | 1/21 (4.8) | Omitted (collinearity) | Omitted (collinearity) |
| Residential type |  |  |  |  |  |  |  |
| Rural | 1.0 (1.3) | 21/38 (55.3) | 17/38 (44.7) | 0/38 (0.0) | 0/38 (0.0) | Ref | Ref |
| Urban | 1.7 (2.7) | 102/194 (52.6) | 81/194 (41.8) | 10/194 (5.2) | 1/194 (0.5) | 3.55 (0.46-27.54) | 4.94 (0.50-48.35) |
| Employment |  |  |  |  |  |  |  |
| Unemployed | 2.4 (2.4) | 9/32 (28.1) | 22/32 (68.8) | 1/32 (3.1) | 0/32 (0.0) | Ref | Ref |
| Employed by someone | 1.4 (2.3) | 36/61 (59.0) | 23/61 (37.7) | 2/61 (3.3) | 0/61 (0.0) | 0.86 (0.19-3.87) | 0.52 (0.88-3.43) |
| Self-employed | 1.5 (2.7) | 78/141 (55.3) | 55/141 (39.0) | 7/141 (5.0) | 1/141 (0.7) | 0.82 (0.21-3.12) | 0.33 (0.06-1.72) |
| Relationship status |  |  |  |  |  |  |  |
| Not in a relationship | 2.3 (3.0) | 32/85 (37.7) | 46/85 (54.1) | 6/85 (7.1) | 1/85 (1.2) | Ref | Ref |
| In a relationship | 1.2 (2.2) | 92/151 (60.9) | 55/151 (36.4) | 4/151 (2.7) | 0/151 (0.0) | 0.38 (0.15-0.98)* | 0.32 (0.10-1.02) |
| Number of children |  |  |  |  |  |  |  |
| No children | 1.1 (1.4) | 14/25 (56.0) | 11/25 (44.0) | 0/25 (0.0) | 0/25 (0.0) | Ref | Ref |
| 1-3 children | 1.7 (2.8) | 79/148 (53.4) | 60/148 (40.5) | 8/148 (5.4) | 1/148 (0.7) | 2.34 (0.66-8.35) | 3.13 (0.67-14.66) |
| 4+ children | 1.6 (2.4) | 30/61 (49.2) | 29/61 (47.5) | 2/61 (3.3) | 0/61 (0.0) | Omitted (collinearity) | Omitted (collinearity) |
| Care status |  |  |  |  |  |  |  |
| Lost to care | 1.6 (2.0) | 53/109 (48.6) | 55/109 (50.5) | 1/109 (0.9) | 0/109 (0.0) | Ref | Ref |
| In care | 1.7 (3.0) | 73/127 (55.9) | 46/127 (36.2) | 9/127 (7.1) | 1/127 (0.8) | 1.96 (0.72-5.34) | 1.84 (0.55-6.23) |
| Duration of living with HIV |  |  |  |  |  |  |  |
| <5 years | 1.3 (2.1) | 76/126 (60.3) | 47/126 (37.3) | 3/126 (2.4) | 0/126 (0.0) | Ref | Ref |
| 5-10 years | 1.7 (2.5) | 21/45 (46.7) | 22/45 (48.9) | 2/45 (4.4) | 0/45 (0.0) | 0.93 (0.24-3.59) | 0.82 (0.19-3.57) |
| >10 years | 2.4 (3.2) | 24/62 (38.7) | 32/62 (51.6) | 5/62 (8.1) | 1/62 (1.6) | 1.65 (0.59-4.67) | 2.42 (0.63-9.37) |

Table 3. Sensitivity analysis results: Adjusted prevalence ratios (aPRs) for distress by subgroups from modified Poisson regression

|  | Prevalence ratio (95% CI) for moderate to severe scores |
| --- | --- |
| CTC facility |  |
| Dodoma Regional Referral Hospital | Ref |
| Makole Health Centre | 0.39 (0.23-0.64)* |
| Sex |  |
| Female | Ref |
| Male | 1.07 (0.62-1.86) |
| Age |  |
| 18-25 | Ref |
| 26-35 | 1.16 (0.55-2.44) |
| 36-45 | 0.73 (0.32-1.66) |
| 46-55 | 0.61 (0.28-1.32) |
| 56+ | 0.67 (0.30-1.49) |
| Residential type |  |
| Rural | Ref |
| Urban | 0.91 (0.56-1.48) |
| Employment |  |
| Unemployed | Ref |
| Employed by someone | 0.85 (0.44-1.63) |
| Self-employed | 0.65 (0.37-1.15) |
| Relationship status |  |
| Not in a relationship | Ref |
| In a relationship | 0.55 (0.34-0.90)* |
| Number of children |  |
| No children | Ref |
| 1-3 children | 0.84 (0.50-1.42) |
| 4+ children | 0.67 (0.34-1.31) |
| Care status |  |
| Lost to care | Ref |
| In care | 1.95 (1.16-3.28)* |
| Duration of living with HIV |  |
| <5 years | Ref |
| 5-10 years | 0.80 (0.42-1.50) |
| >10 years | 1.64 (0.96-2.80) |

* p<0.05
